# Malaria Host Candidate Genes Validated by Association With Current, Recent, and Historical Measures of Transmission Intensity

**DOI:** 10.1093/infdis/jix250

**Published:** 2017-05-25

**Authors:** Nuno Sepúlveda, Alphaxard Manjurano, Susana G. Campino, Martha Lemnge, John Lusingu, Raimos Olomi, Kirk A. Rockett, Christina Hubbart, Anna Jeffreys, Kate Rowlands, Taane G. Clark, Eleanor M. Riley, Chris J. Drakeley, the MalariaGEN Consortium

**Affiliations:** 1 London School of Hygiene and Tropical Medicine,; 2 Wellcome Trust Sanger Institute, Hinxton, and; 3 Wellcome Trust Centre for Human Genetics, University of Oxford, United Kingdom;; 4 Centre of Statistics and Applications, University of Lisbon, Portugal;; 5 Joint Malaria Programme, Kilimanjaro Christian Medical Centre, Moshi, and; 6 National Institute for Medical Research, Dar es Salaam, Tanzania

**Keywords:** Malaria, SNP, transmission intensity, genetic association.

## Abstract

**Background.:**

Human malaria susceptibility is determined by multiple genetic factors. It is unclear, however, which genetic variants remain important over time.

**Methods.:**

Genetic associations of 175 high-quality polymorphisms within several malaria candidate genes were examined in a sample of 8096 individuals from northeast Tanzania using altitude, seroconversion rates, and parasite rates as proxies of historical, recent, and current malaria transmission intensity. A principal component analysis was used to derive 2 alternative measures of overall malaria propensity of a location across different time scales.

**Results.:**

Common red blood cell polymorphisms (ie, hemoglobin S, glucose-6-phosphate dehydrogenase, and α-thalassemia) were the only ones to be associated with all 3 measures of transmission intensity and the first principal component. Moderate associations were found between some immune response genes (ie, *IL3* and *IL13*) and parasite rates, but these could not be reproduced using the alternative measures of malaria propensity.

**Conclusions.:**

We have demonstrated the potential of using altitude and seroconversion rate as measures of malaria transmission capturing medium- to long-term time scales to detect genetic associations that are likely to persist over time. These measures also have the advantage of minimizing the deleterious effects of random factors affecting parasite rates on the respective association signals.

## Summary

Genetic association within several malaria candidate genes was examined in 24 villages of northeast Tanzania, using different measures of malaria transmission intensity. We demonstrate that the classic hemoglobinopathies were associated with measures of transmission intensity that had longer time scales.

Malaria caused by the parasite *Plasmodium falciparum* continues to have a significant impact on public health in tropical areas of the world. Although major advances have been made in malaria control, there is increasing parasite drug resistance and mosquito insecticide resistance, and no fully efficacious vaccine exists. A better understanding of the factors associated with immunity to and protection from malaria may contribute to the development of vaccines and other therapies. It is widely recognized that malaria has exerted strong genetic selection on the human genome [1]. Mutations leading to sickle cell trait (hemoglobin S [HbS]) were central to the hypothesis, proposed by Haldane in the 1940s [2], that the high prevalence of hemoglobinopathies in southern European and African populations was due to the protection against malaria in carriers of these traits.

This hypothesis has subsequently been validated for several hemoglobinopathies, indirectly by the concordance between the prevalence of malaria parasites and frequencies of mutated alleles [3, 4] and directly through protection from disease in carriers [4–7]. The different geographic distributions of HbS, α-thalassemia, glucose-6-phosphate dehydrogenase (G6PD) deficiency, ovalocytosis, and the Duffy-negative blood group are examples of the general principle that different genetic variants have arisen and been selected in different populations (see [8] for a review). The most striking example is the β-globin *HBB* gene, in which 3 different coding single-nucleotide polymorphisms (SNPs) confer protection against malaria: Glu6Val (HbS), Glu6Lys (hemoglobin C), and Glu26Lys (hemoglobin E). The mechanisms by which the various hemoglobinopathies protect individuals from malaria are not fully understood [9, 10].

There is increasing evidence that other traits, particularly those relevant to immunity and inflammation, may also be selected by malaria infection; these include polymorphisms in genes encoding tumor necrosis factor (*TNF*, MHC class III region, reviewed in [11]; Toll-like receptors (*TLR4*, *TLR9*) [12]; CD40 ligand (*CD40L*) [13]; interferon γ (*IFNG*), reviewed in [14]; and nitric oxide synthase type 2 (*NOS2*A), reviewed in [15]). These associations are consistent with the observation that disease severity (and thus malaria-related mortality risk) is associated with the strength of the inflammatory response to malaria infection [16].

The bulk of the evidence linking genotype to malaria susceptibility stems from studies of severe and/or complicated disease ,where the genetic association might be the strongest owing to the close temporal relationship between genotype, consequence (infection or inflammation), and effect (disease or death). Conversely, genetic traits that reduce the risk of becoming patently infected with malaria [17], such as those operating at the pre-erythrocytic stage of infection, are less likely to be detected because their effect is too small or because controls tend to be drawn from those with patent but nonsevere infection. However, these traits may become apparent in large population-based studies. Landmark studies by Flint et al [4, 5] in Melanesia were the first to use the natural cline in malaria, associated with latitude and altitude, to demonstrate a positive correlation between malaria transmission and the prevalence of α-thalassemia, an observation replicated in northeast Tanzania, where a negative correlation was observed between altitude (as a proxy for malaria transmission) and the prevalence of HbS and α-thalassemia heterozygotes [3]. Integrated approaches have now shown that the frequency of different genetic polymorphisms varies with malaria transmission intensity at a regional and at a global scale [18–20].

Malaria transmission intensity can be defined in a variety of ways, including the incidence of clinical disease, the frequency of being bitten by infected mosquitoes, and the environmental suitability for transmission [21]. These definitions are linked to different epidemiological measures that capture distinct aspects of transmission, including risk of exposure (geographic, climatic, and mosquito-related variables), actual exposure (serological data), infection (parasitological variables) and disease (clinical data). These measures are associated with differing time scales, ranging from generations (for risk) to years (for exposure), months or weeks (for infection) or days (for disease). In turn, these different measures may be associated with different genetic markers, depending on the clinical or parasitological consequences of the respective genetic effects. In the current study, we combine a candidate-gene approach with different measures of malaria transmission to investigate malaria genetic associations in populations living in northeast Tanzania.

## METHODS

### Ethical Approvals

Ethical approval was obtained from the London School of Hygiene and Tropical Medicine, Kilimanjaro Christian Medical College in Tanzania, and the Tanzanian National Medical Research Institute.

### Study Sites

**Figure 1.**
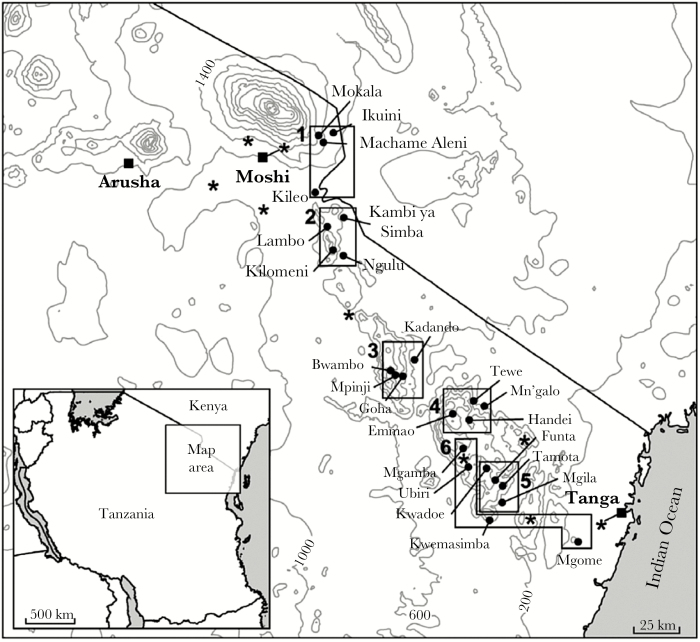
Map of study sites and the corresponding transects: Rombo (transect 1), North Pare (transect 2), South Pare (transect 3), West Usambara 1 (transect 4), West Usambara 2 (transect 5), and West Usambara 3 (transect 6). Locations of 8 meteorological stations are also shown *(asterisks*).

The study was conducted in the Kilimanjaro and Tanga regions of northeast Tanzania. Cross-sectional age-stratified malariometric surveys, conducted in 24 villages in 6 transects (Figure 1 and Table 1) after the short rainy season in November 2001 and again after the long rains the following June (2002), as described elsewhere [22], generated between 194 and 435 samples from unique individuals 1–45 years old in each village. To control for population structure, each transect was selected to approximately represent a specific ethnic group (Table 1).

**Table 1.** Baseline Information on Different Malaria Transmission Measures for the 24 Villages Considered in the Study

**Transect and Village Name (sample size** **)^a^**	**Altitude, Mean (Range), m**	**Serology**		**Parasite Rate, %**	
		**Seroprevalence, %**	**SCR^b^**	**Overall**	**Age 2–10 y**
Kilimanjaro region					
Rombo (Wachaga, 80%)					
Mokala (413)	1702 (1623–1788)	20.7	0.027	4.4	5.6
Machame Aleni (249)	1421 (1380–1482)	26.2	0.038	1.3	0.9
Ikuini (363)	1160 (1118–1215)	24.2	0.033	12.3	11.0
Kileo (247)	723 (721–724)	78.6	0.343	6.6	8.6
North Pare (Wapare, 92%)					
Kilomeni (351)	1556 (1384–1745)	36.1	0.044	3.4	1.0
Lambo (295)	1187 (1146–1231)	62.7	0.147	2.5	3.9
Ngulu (405)	831 (798–863)	93.4	0.765	6.2	8.5
Kambi ya Simba (281)	745 (716–767)	90.9	0.626	10.1	13.8
South Pare (Wapare, 90%)					
Bwambo (422)	1598 (1564–1643)	25.5	0.033	3.5	3.4
Mpinji (386)	1445 (1307–1667)	35.3	0.054	2.8	3.2
Goha (408)	1162 (1079–1228)	59.8	0.144	11.4	14.2
Kadando (414)	528 (525–531)	79.7	0.333	23.4	28.3
Tanga region					
West, Usambara 1 (Wasambaa, 88%)					
Emmao (199)	1845 (1810–1872)	17.3	0.019	3.7	0.0
Handei (417)	1425 (1372–1517)	68.1	0.209	27.9	32.1
Tewe (372)	1049 (965–1126)	69.9	0.205	34.4	35.2
Mng’alo (398)	416 (406–455)	90.9	0.802	49.2	69.3
West, Usamabara 2 (Wasambaa, 88%)					
Kwadoe (435)	1523 (1473–1603)	41.6	0.073	8.5	6.5
Funta (315)	1279 (1236–1322)	73.7	0.268	24.7	27.6
Tamota (417)	1176 (1073–1338)	59.3	0.140	24.7	24.8
Mgila (391)	432 (406–455)	86.5	0.543	41.4	55.6
West, Usambara 3 (Wasambaa, 65%)					
Magamba (237)	1685 (1659–1751)	39.4^c^	0.048^c^	3.2	1.0
Ubiri (194)	1216 (1174–1262)	47.0^c^	0.052^c^	16.2	14.8
Kwemasimba (247)	662 (636–691)	64.7^c^	0.103^c^	25.3	32.4
Mgome (240)	196 (165–208)	96.5	0.941	40.2	69.0

Abbreviation: SCR, seroconversion rate.

^a^The major ethnic group and its respective percentage is given parenthetically after each transect name.

^b^SCR was defined as the yearly average frequency at which seronegative individuals became seropositive.

^c^Estimates based on data imputation performed using MICE software.

A finger-prick blood sample was collected from each participant for assessment of parasitemia, antimalarial antibody responses and genotype. Written consent was provided by all participants or by their guardians, and clinical illnesses were treated in accordance with national guidelines.

### Blood Slide Examination

The presence of *P. falciparum* parasites was determined by microscopic examination of Giemsa-stained blood smears. Asexual (blood-stage) parasite density was calculated relative to 200 white blood cells [22]. Each slide was read by 2 independent microscopists, and noncongruent readings were resolved by a third reader.

### Enzyme-Linked Immunosorbent Assays and Seroconversion Rate Calculation

Serological characterization of the samples through enzyme-linked immunosorbent assays and calculation of seroconversion rate (SCR) (ie, the rate at which individuals become seropositive per year) has been published elsewhere [23]. Briefly, indirect enzyme-linked immunosorbent assays were used to detect immunoglobulin G (antibodies to blood-stage malaria antigens merozoite surface protein 1_19_ [K1-Wellcome genotype] and apical membrane antigen 1 [3D7], produced as described elsewhere [24, 25]). Antibody responses in serum from Europeans with no previous exposure to malaria were used to define a cutoff for seropositivity (mean optical density plus 3 standard deviations) for each antigen. Seroprevalence was calculated for each village as the respective proportion of seropositive individuals. SCRs were estimated for each antigen and village using reversible catalytic models, where each individual transits between seronegativity and seropositivity over time under the assumption of stable and constant malaria transmission intensity.

### DNA Extraction and Genotyping

DNA was extracted from the archived, pelleted, blood samples using Nucleon kits (Hologic) according to the manufacturer’s instructions. SNP genotypes were determined by means of primer-extension mass spectrometry using the Sequenom iPlex platform MassARRAY system (Agena Bioscience) at the Wellcome Trust Centre for Human Genetics in Oxford. The α^3.7^-thalassemia deletion was identified using polymerase chain reaction amplification, as described elsewhere [3]. A total of 275 SNP assays were designed incorporating a core set of 65 SNPs (as described elsewhere [26]), plus a further 137 autosomal SNPs selected in genes associated with or described as associated with antibody production [27], and another 73 identified in a previous study [28].

### Statistical Analysis

SNPs were removed from the analysis if monomorphic, if the allele frequency was <1%, if >10% of their genotypes were missing, or if there was evidence of extreme deviation from Hardy-Weinberg equilibrium (*P* value < .001; χ^2^ test). Because the sample and actual population sizes are close in many villages (mostly those in the highlands owing to their geographic isolation), genetic association analyses were conducted using aggregated data from each village: mean altitude, parasite prevalence, SCR, genotype distributions, male-female ratio, and transect of residence. Genotype frequencies of each SNP were summarized for each village and then rescaled using the log-additive transformation [29], with the heterozygous genotype frequencies used as reference, that is,

$$p_{1ij}=log\frac{f_{AA,ij}}{f_{AB,ij}}\;and\;p_{2ij}=log\frac{f_{BB,ij}}{f_{AB,ij}}\text{,i = 1,}...\text{,275, j = 1,}...\text{,24,}$$

where *f*_AA,*ij*_, *f*_AB,ij_, and *f*_BB,*ij*_ are the genotype frequencies of AA, AB, and BB, respectively; A and B are the reference and alternative allele of SNP *i*, calculated for village *j*. For SNP where the frequency *f*_BB_ was too small (eg, rs334 related to HbS), the following data transformation was used:

$$p_{1ij}=\text{log}\frac{f_{AA,ij}}{f_{AB,ij}+f_{BB,ij}}.$$

Missing genotype frequencies of the α-thalassemia locus from 11 villages [30] plus missing SCRs from 3 other villages (Table 1) were jointly imputed via multiple imputation using chained equations [31]. Multiple imputation was performed at the village level and not at the individual level as described elsewhere [30]. Altitude, log odds of parasite rate, sex log ratio, and transect of residence were included in the imputation models as fixed covariates. Postimputation estimates and standard errors were based on 100 imputed data sets and determined as described elsewhere [32].

Evidence for genetic association of a given SNP with each malaria transmission measure was assessed by multivariate linear regression based on a multivariate normal distribution, using the log-additive genotype distributions as dependent variables and the logarithm of the male-female ratio and the transect of residence and the different malaria transmission measures as covariates. Models were tested with SCR and altitude in linear or in log, choosing the scale that provided the best fit for the data. Log odds of parasite prevalence were used in the analysis. The Wilks likelihood ratio was then used to compare models with or without a given malaria transmission measure. For consistency, –log_10_(*P* value) was considered a measure of statistical significance. High values of this measure provided evidence for an association between a given genetic marker and the respective transmission intensity measure. Because data refer to a set of candidate genes for which there is a priori evidence of association with malaria susceptibility, a significance level of 1% (eg, 2 for –log_10_[*P* value]) was used in each individual association test. Finally, the fitted regression models were statistically checked by residual analyses (eg, normality checks or trends in the residuals).

A principal component analysis of altitude, SCR, and parasite rate enabled the derivation of alternative “latent” measures of malaria transmission. Genetic association was assessed again, as described above. Association analyses involving X chromosome SNPs were performed on male and female data separately. The underlying false discovery rates were finally estimated for the different analyses (Supplementary Table S1). These rates ranged from 0.08 to 0.19; thus, one could not rule out that some of the detected association signals, namely, those close to the significance threshold, were due to chance. All analyses were carried out with R software, using 2 packages: MICE (for data imputation) and Genetics (for genetic analysis) (see *https://cran.r-project.org/*).

## RESULTS

A total of 8241 samples were genotyped for an initial set of 275 SNPs by Sequenom technology. After implementation of quality control protocols, data from 8096 individuals across 175 high-quality SNPs were available for the final analysis. For α^3.7^-thalassemia, 2997 samples were successfully genotyped from 13 villages (Mgome in Tanga and 4 villages each from Kilimanjaro, South Pare, and West Usambara 2), and data imputation was performed in the remaining 11 villages.

### Association of Sickle Cell and α-Thalassemia Traits and Point Mutations in *CD36* and *G6PD* With Historical, Recent, and Current Measures of Malaria Transmission

Genetic variation analysis confirmed previous observations of significant inverse gradients of α-thalassemia and HbS and altitude (−log_10_[*P* value], 5.30 and 4.33, respectively; Table 2 and Supplementary Figure S1*A*) [3]. The odds of noncarrier versus HbS carrier increased by approximately 0.16 per 100 m increase in altitude, whereas the same odds of the noncarriers of the α-thalassemia trait increased by approximately 0.08 per 100 m using data imputation. Similar genetic effects for the α-thalassemia trait were obtained for the complete data of 13 villages but with increased standard errors. Strong associations were found between genotype frequency of these 2 traits and SCR and parasite prevalence (−log_10_[*P* value], >2.61; Table 2 and Supplementary Figure S1*B* and S1*C*).

Significant associations were detected between all malaria transmission measures and SNP rs3211938 in *CD36* (−log_10_[*P* value], >2.58; Table 2 and Supplementary Figure S1) and several point mutations within the X-linked *G6PD* locus (Table 3 and Supplementary Figure S2). For *G6PD*, the associations were most pronounced among female participants and for current transmission intensity (parasite prevalence) compared with historical or recent measures of malaria transmission. These associations are in agreement with recent findings from the same area, showing that heterozygous women are more protected against severe malaria than men [33].

**Table 3.** Significant Genetic Associations Between Malaria Transmission Measures and X-linked G6PD SNPs Using −Log_10_(*P* value) as an Association Measure With a Cutoff of 2^a^

**Measure and SNP**	**Position**	**Alleles A/B**	**Scale**	−**log**_**10**_**(*P* Value**)	**Estimates (SE**)		
					**log *f***_**AA**_ **/*f***_**AB**_	**log *f***_**BB**_ **/*f***_**AB**_	**log *f***_**A**_ **/*f***_**B**_
Altitude, female participants							
rs28470352	153753490	T/A	Linear	3.18	0.023 (0.006)	−0.008 (0.031)	NA
rs5986990	153761628	G/A	Linear	3.36	0.022 (0.006)	−0.007 (0.031)	NA
rs2515905	153762075	G/A	Log	2.51	0.214 (0.075)	0.379 (0.647)	NA
rs2515904	153762771	G/C	Log	2.76	0.204 (0.059)	0.245 (0.651)	NA
rs1050829	153763492	T/C	Linear	2.16	0.018 (0.007)	−0.009 (0.033)	NA
rs762516	153764663	C/T	Log	2.61	0.201 (0.061)	0.251 (0.638)	NA
Altitude, male participants							
rs1050829	153763492	T/C	Linear	2.05	NA	NA	0.027 (0.010)
SCR, female participants^b^							
rs28470352	153753490	T/A	Log	2.33	−0.069 (0.030)	−0.064 (0.128)	NA
rs5986990	153761628	G/A	Log	2.53	−0.062 (0.029)	−0.095 (0.127)	NA
rs2515905	153762075	G/A	Linear	2.12	−0.345 (0.176)	−0.939 (1.372)	NA
rs2515904	153762771	G/C	Linear	3.22	−0.430 (0.128)	−0.685 (1.380)	NA
rs1050828	153764217	C/T	Linear	2.85	−0.591 (0.195)	−0.603 (1.136)	NA
rs762516	153764663	C/T	Linear	3.00	−0.422 (0.133)	−0.612 (1.354)	NA
SCR, male participants^b^							
rs1050828	153764217	C/T	Log	3.24	NA	NA	−0.114 (0.050)
Parasite rate, female participants							
rs28470352	153753490	T/A	Log odds	5.04	−0.109 (0.029)	0.211 (0.135)	NA
rs2230037	153760654	G/A	Log odds	2.43	0.170 (0.052)	0.019 (0.061)	NA
rs5986990	153761628	G/A	Log odds	5.17	−0.104 (0.027)	0.154 (0.139)	NA
rs2515905	153762075	G/A	Log odds	2.57	−0.107 (0.044)	0.231 (0.363)	NA
rs2515904	153762771	G/C	Log odds	4.14	−0.113 (0.034)	0.288 (0.360)	NA
rs1050829	153763492	T/C	Log odds	4.66	−0.097 (0.030)	0.221 (0.141)	NA
rs1050828	153764217	C/T	Log odds	3.88	−0.070 (0.062)	0.379 (0.288)	NA
rs762516	153764663	C/T	Log odds	3.84	−0.112 (0.035)	0.289 (0.353)	NA
Parasite rate, male participants							
rs28470352	153753490	T/A	Log odds	2.96	NA	NA	0.148 (0.051)
rs1050829	153763492	T/C	Log odds	4.56	NA	NA	0.143 (0.036)
rs1050828	153764217	C/T	Log odds	4.35	NA	NA	0.125 (0.059)
rs762516	153764663	C/T	Log odds	3.12	NA	NA	0.164 (0.060)

Abbreviations: NA, not applicable; SCR, seroconversion rate; SE, standard error; SNP, single-nucleotide polymorphism.

^a^SNPs were deemed significant if −log10(*P* value) was >2.

^b^Results based on data imputation performed using MICE software.

### Association Between Variation Within Immune Response Genes and Current Malaria Transmission Levels

Variation in frequency of a number of SNPs located within immune response associated genes is statistically associated with parasite rate (Table 2). These SNPs included 5 within *DDC* (which encodes dopa decarboxylase/aromatic L-amino acid decarboxylase, an essential component of the dopamine/serotonin/tryptamine pathway); SNPs in genes encoding interleukin 3, interleukin 13, and CTLA-4; and 2 SNPs in *PDLIM4* (believed to play a role in bone development and homeostasis). Note, however, that the signals of association are moderate compared with those for sickle cell, α-thalassemia, and G6PD.

**Table 2.** Significant Genetic Associations Between Malaria Transmission Measures and Autosomal SNPs Using −log_10_(*P* value) as an Association Measure With a Cutoff of 2^a^

**Measure and SNP**	**Chr**	**Position**	**Gene**	**Alleles A/B**	**Scale**	−**log**_**10**_**(*P* Value**)	**Estimates (SE**)	
							**log *f***_**AA**_ **/*f***_**AB**_	**log *f***_**BB**_ **/*f***_**AB**_
Altitude								
rs11575518	7	50535681	*DDC*	G/A	Log	2.00	0.821 (0.551)^b^	−0.166 (0.087)^b^
rs3211938	7	80300449	*CD36*	T/G	Log	3.73	0.575 (0.153)^b,c^	NA
rs334	11	5248232	*HBB*	A/T	Linear	4.33	0.162 (0.032)^b,c^	NA
α-Thalassemia^d^	16	222500	*HBA2*	α^+^/α^−^	Linear	3.28	0.078 (0.015)^b^	−0.105 (0.153)^b^
α-Thalassemia^e^	16	222500	*HBA2*	α^+^/α^−^	Linear	5.30	0.081 (0.019)^b^	−0.071 (0.027)^b^
SCR								
rs3211938	7	80300449	*CD36*	T/G	Log	2.59	−0.241 (0.084)^c^	NA
rs334	11	5248232	*HBB*	A/T	Log	3.48	−0.591 (0.151)^c^	NA
α-Thalassemia^d^	16	222500	*HBA2*	α^+^/α^−^	Log	5.58	−0.337 (0.086)	0.443 (0.709)
α-Thalassemia^e^	16	222500	*HBA2*	α^+^/α^−^	Linear	5.30	−0.315 (0.081)	0.294 (0.113)
Parasite rate								
rs31481	5	131397202	*IL3*	G/A	Log odds	2.14	−0.097 (0.032)	−0.091 (0.116)
rs3900945	5	131592870	*PDLIM4*	C/T	Log odds	2.49	−0.073 (0.047)	0.003 (0.091)
rs10463891	5	131597392	*PDLIM4*	G/A	Log odds	2.65	−0.080 (0.046)	−0.031 (0.082)
rs2069744	5	131994669	*IL13*	C/T	Log odds	2.20	−0.025 (0.043)	0.017 (0.052)
rs2242665	6	31839309	*CTLA4*	G/A	Log odds	2.04	−0.080 (0.072)	0.038 (0.049)
rs11983581	7	50532888	*DDC*	A/T	Log odds	2.54	0.470 (0.309)	0.079 (0.053)
rs11982772	7	50533062	*DDC*	A/C	Log odds	2.84	0.077 (0.047)	0.467 (0.310)
rs11575527	7	50534327	*DDC*	G/C	Log odds	2.59	0.084 (0.053)	0.485 (0.308)
rs11575522	7	50535395	*DDC*	C/T	Log odds	2.63	0.076 (0.051)	0.476 (0.305)
rs11575518	7	50535681	*DDC*	G/A	Log odds	2.83	0.483 (0.308)	0.083 (0.050)
rs3211938	7	80300449	*CD36*	T/G	Log odds	3.19	−0.235 (0.102)^c^	NA
rs334	11	5248232	*HBB*	A/T	Log odds	2.61	−0.610 (0.181)^c^	NA
α-Thalassemia^d^	16	222500	*HBA2*	Α^+^/α^−^	Log odds	3.39	−0.390 (0.081)	1.098 (0.678)
α-Thalassemia^e^	16	222500	*HBA2*	Α^+^/α^−^	Log odds	5.78	−0.377 (0.086)	0.288 (0.133)

Abbreviations: Chr, chromosome; NA, not applicable; SCR, seroconversion rate; SE, standard error; SNP, single-nucleotide polymorphism.

^a^SNPs were deemed significant if −log_10_(*P* value) was >2.

^b^Estimates per 100-m increase in altitude.

^c^Estimates based on log *f*_AA_/(*f*_AB_+ *f*_BB_).

^d^Results based on complete data (13 villages).

^e^Results based on data imputation performed using MICE software.

### Association of Alternative Measures of Malaria Propensity With Genetic Variation

**Figure 2.**
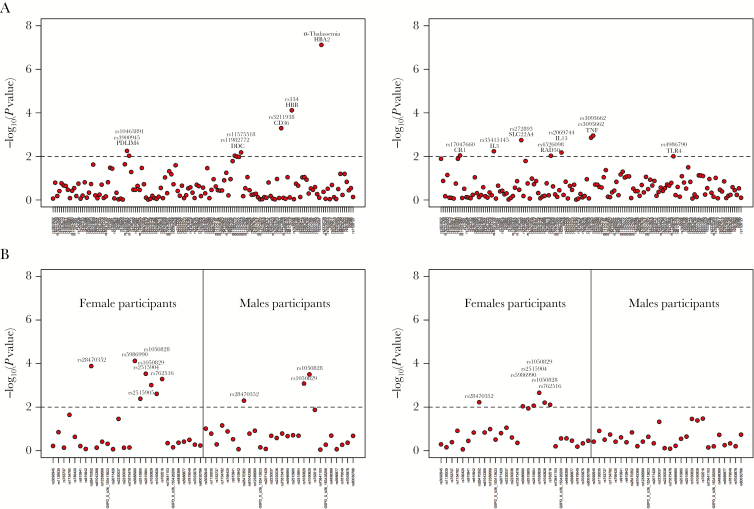
Association between single-nucleotide polymorphisms (SNPs) and 2 principal components generated from summary data of the 3 malaria transmission measures for each village. *A,* Association of autosomal SNPs and each principal component. *B,* Association of X-linked SNPs and each principal component. Dashed lines represent the cutoff set for significant association. Specifically, a given genetic association was deemed significant for SNPs when the −log_10_(*P* value) was >2 (ie, *P* < .01).

Although altitude, SCR and parasite prevalence capture different time scales of malaria transmission, these measures were highly correlated with each other (Supplementary Table S2). Altitude was inversely correlated with both SCR and parasite rate (*R*^2^ < −0.688) whereas SCR was positively correlated with parasite rate (*R*^2^ = 0.568). These correlations were further explored using 2 principal components that accounted for 95% of the total variation of these measures (Supplementary Table S2). These principal components could be interpreted as 2 independent measures of malaria propensity of a village across historical, recent, and current infection. The respective derived data were then tested for genetic association (Figure 2 and Table 4). HbS, α-thalassemia, CD36, several SNPs in the *G6PD* locus either in female or male participants, and borderline associations with genetic markers at the *DDC* and *PDLIM4* loci were associated with the first principal component. For the second principal component, there was an enrichment of immune response genes (*IL3, TNF, TLR4,* and *CR1*), although the corresponding associations were not strong (2.00 < −log_10_(*P* value) < 3.00). Of note, the genetic variation at the *G6PD* locus could be explained by both principal components.

**Table 4.** Detected Associations Between the SNPs and the First 2 PCs Using A Statistically Significant Cutoff of 2 for −log_10_(*P* value)

**Measure, SNP**	**Chr**	**Position**	**Gene**	**Alleles A/B**	−**log**_**10**_**(*P* Value**)	**Estimates (SE**)		
						**log *f***_**AA**_ **/*f***_**AB**_	**log *f***_**BB**_ **/*f***_**AB**_	**log *f***_**A**_ **/*f***_**B**_
1st PC, overall								
rs3900945	5	131592870	*PDLIM4*	C/T	2.25	−0.063 (0.030)	0.040 (0.065)	NA
rs10463891	5	131597392	*PDLIM4*	G/A	2.03	−0.061 (0.030)	0.019 (0.055)	NA
rs11982772	7	50533062	*DDC*	G/A	2.03	−0.049 (0.032)	0.317 (0.207)	NA
rs11575518	7	50535681	*DDC*	G/A	2.18	0.326 (0.206)	0.054 (0.034)	NA
rs3211938	7	80300449	*CD36*	T/G	3.30	−0.189 (0.063)^a^	NA	NA
rs334	11	5248232	*HBB*	A/T	4.12	−0.485 (0.102)^a^	NA	NA
α-Thalassemia	16	222500	*HBA2*	α^+^/α^−^	7.12	−0.262 (0.056)	0.226 (0.085)	NA
1st PC, female participants								
rs28470352	X	153753490	*G6PD*	T/A	3.88	−0.069 (0.020)	0.034 (0.097)	NA
rs5986990	X	153761628	*G6PD*	G/A	4.12	−0.064 (0.019)	0.012 (0.097)	NA
rs2515905	X	153762075	*G6PD*	G/A	2.39	−0.067 (0.030)	−0.128 (0.244)	NA
rs2515904	X	153762771	*G6PD*	G/C	3.53	−0.074 (0.023)	−0.089 (0.245)	NA
rs1050829	X	153763492	*G6PD*	T/C	3.01	−0.056 (0.022)	0.037 (0.101)	NA
rs1050828	X	153764217	*G6PD*	C/T	2.61	−0.063 (0.040)	0.012 (0.203)	NA
rs762516	X	153764663	*G6PD*	C/T	3.29	−0.072 (0.024)	−0.086 (0.240)	NA
1st PC, male participants								
rs28470352	X	153753490	*G6PD*	T/A	2.30	NA	NA	−0.0812 (0.0370)
rs1050829	X	153763492	*G6PD*	T/C	3.08	NA	NA	−0.0766 (0.0283)
rs1050828	X	153764217	*G6PD*	C/T	3.50	NA	NA	−0.0838 (0.0392)
2nd PC, overall								
rs17047660	1	207782856	*CR1*	A/G	2.04	0.135 (0.095)	0.027 (0.219)	NA
rs35415145	5	131396406	*IL3*	C/T	2.24	0.070 (0.229)^a^	NA	NA
rs272893	5	131663062	*SLC22A4*	A/G	2.75	−0.214 (0.235)	−0.098 (0.073)	NA
rs4526098	5	131892979	*RAD50*	G/A	2.03	−0.225 (0.133)	0.026 (0.121)	NA
rs2069744	5	131994669	*IL13*	C/T	2.18	−0.085 (0.111)	−0.133 (0.127)	NA
rs361525	6	31543101	*TNF*	G/A	2.86	0.258 (0.155)	−0.482 (0.903)	NA
rs3093662	6	31544189	*TNF*	A/G	2.95	0.138 (0.128)	−0.672 (0.476)	NA
rs4986790	9	120475302	*TLR4*	A/G	2.01	0.285 (0.186)	1.277 (1.432)	NA
2nd PC, female participants								
rs28470352	X	153753490	*G6PD*	T/A	2.22	−0.046 (0.098)	0.983 (0.266)	NA
rs5986990	X	153761628	*G6PD*	G/A	2.04	−0.056 (0.091)	0.893 (0.289)	NA
rs2515904	X	153762771	*G6PD*	G/C	2.06	−0.005 (0.109)	2.484 (0.706)	NA
rs1050829	X	153763492	*G6PD*	T/C	2.66	−0.084 (0.093)	1.011 (0.283)	NA
rs1050828	X	153764217	*G6PD*	C/T	2.21	0.177 (0.156)	2.119 (0.571)	NA
rs762516	X	153764663	*G6PD*	C/T	2.11	−0.018 (0.110)	2.434 (0.699)	NA

Abbreviations: Chr, chromosome; NA, not applicable; PC, principal component; SE, standard error; SNP, single-nucleotide polymorphism.

^a^Estimates based on log *f*_AA_/(*f*_AB_+*f*_BB_).

## DISCUSSION

Geographic variation in the frequency of hemoglobinopathies provided the first evidence that these traits might protect against disease or death caused by malaria. They tend to be rare in areas of low malaria transmission and more common in areas of higher transmission. In this study, we have used local altitude-dependent variations in malaria transmission intensity to both confirm and identify additional human genetic polymorphisms relevant for individuals living in the study area.

The present study focused on a set of genetic polymorphisms previously implicated in resistance to malaria. Replication of many of these associations has been difficult because, although some polymorphisms are likely to be genuinely (and causally) associated with malaria resistance, other associations may have arisen by chance (eg, in small studies or owing to population structure or instability of malaria infection measures) or may be closely linked to causal variants in some populations but not in others [34]. Possible population structure was controlled by a study design comprising transects that represented specific ethnic groups. Well-matched age distributions and sample sizes ensured similar precision of the aggregated data across villages.

The use of altitude and SCR in the genetic analysis reduced the chance of detecting sporadic associations due to the intrinsic instability of the parasite rate in estimating the underlying malaria transmission intensity. Altitude, through the effect of temperature on sporogonic development of malaria parasites in the mosquito, is a stable proxy for historical, recent, and ongoing malaria exposure stretching back to the original settlement of these villages some 4000–5000 years ago [35].

SCR is a more direct measure of exposure to infection but estimated from a mathematical model that assumed constant and stable malaria transmission intensity over time. Such a model, although fitting the data well, might mask possible slow (linear) trends in disease transmission intensity occurring over time [36]. Estimates of SCR should be then seen as averages of recent transmission intensities. This averaging effect might reduce the power of identifying genetic associations with more subtle effects. Notwithstanding this limitation, SCR analysis suggests that malaria transmission intensities in the study villages could be considered approximately stable for about 40 years before sampling in 2001 [23], and probably before this [37]. The same likely holds true for the resulting genetic association due to malaria, given that there have been no major migrations or admixture events in the recorded history of this area

The approach presented here was validated by strong genetic associations of HbS, α-thalassemia, and G6PD deficiency, each of which showed marked variation with the different malaria transmission intensities. As anticipated, geographic variation in the frequency of sickle cell trait and α-thalassemia was most highly correlated with altitude and is thus a stable marker of malaria exposure over many generations. Moreover, both these traits were also highly associated with SCR and parasite prevalence, suggesting stable genetic associations over time. Interestingly, G6PD deficiency was most significantly associated with parasite rate (stronger in female than in male participants) rather than with altitude or SCR. This may reflect the fact that mutations in this gene protect from severe clinical outcomes rather than infection per se [2].

Given the geographic variation in the prevalence of classic malaria resistance traits and their strong association with transmission intensity, it was surprising that relatively few of the other “malaria-associated” polymorphisms showed any such variation and/or association. Of the 70 genes analyzed, only 6 (*CD36, DDC, IL3, IL13, CTLA4,* and *PDLIM4*) showed any direct association with malaria transmission measures. Interestingly, polymorphisms in all of these loci were significantly associated with parasite prevalence, but only 2 (*CD36* and *DDC*) were associated with altitude and only 1 (*CD36*) with SCR. Although located in the same chromosome, these genes are not linked to each other. Three other loci (*CR1, TNF,* and *TLR4*) showed borderline associations with the principal component 2 but not with any particular measure of malaria transmission.

Village-level prevalence of the *CD36* polymorphism (rs3211938) was associated with all 3 transmission intensity measures and with the principal component representing putative long-term effects of malaria exposure. CD36 is a ubiquitously expressed scavenger receptor and a major receptor for the *Plasmodium falciparum* erythrocyte membrane protein 1 family of erythrocyte surface proteins, responsible for sequestration and rosetting of malaria-infected red blood cells [38]. Although an early study suggested an association between *CD36* mutations and susceptibility to severe malaria [39], this link has not been substantiated, and an extensive multiple-country analysis indicated that the high prevalence of the rs3211938 polymorphism in African populations is mostly likely maintained by factors other than malaria [40]. However, the consistent association of rs3211938 with 3 different measures of malaria transmission intensity suggests that *CD36* may indeed contribute to protection against malaria, but perhaps through its role in macrophages as a nonopsonic mediator of phagocytosis or through its role in hemostasis and thrombosis, rather than as an endothelial receptor for infected erythrocytes.

Several linked polymorphisms in the *DDC* locus were associated with parasite prevalence and the principal component pertaining to variation between intermediate and current malaria exposure. This result is agreement with 2 recent studies from Tanzania [28] and Gambia [41] that detected a possible role for *DDC* in severe malaria. Because these various polymorphisms are all in linkage disequilibrium with each other, the corresponding genetic effect might reflect haplotypes protecting from malaria, as demonstrated for the G6PD locus in a study from Tanzania [33]. A detailed analysis of individual haplotype associations was beyond the scope of the current work.

Genetic variations in several immune-response genes (*CR1, TNF, TLR4, IL3, IL13,* and *CTLA4*), as well as in *RAD50, SLC22A4,* and *PDLIM4,* were found to be moderately associated with parasite prevalence and the alternative measure of current transmission intensity based on the second principal component. Because these polymorphisms were associated only with these measures of transmission intensity, one can question their possible replication in future studies. In this regard, parasite rate can be affected by the study design used, time of sampling, seasonal effects on malaria transmission, and sensitivity/specificity of the diagnostic test. Therefore, this measure should be considered less robust than altitude and SCR, which capture longer time scales of transmission intensity.

In summary, genetic associations concerning common red blood cell polymorphisms were confirmed using different measures of malaria transmission intensities. The remaining associations would seem less robust and less likely to be replicated, because they are related to parasite rate and an alternative measure of current transmission intensity. The adopted approach therefore shows the advantage of finding genetic associations that are likely to persist over time.

## Supplementary Data

Supplementary materials are available at *The Journal of Infectious Diseases* online. Consisting of data provided by the authors to benefit the reader, the posted materials are not copyedited and are the sole responsibility of the authors, so questions or comments should be addressed to the corresponding author.

## Supplementary Material

Supplementary_Figure_1

Supplementary_Figure_2

Supplementary_Figure_3

Supplementary_Table_1

Supplementary_Table_2

## Notes

***Acknowledgments.*** We thank all our colleagues within the Joint Malaria Programme, a collaboration between the London School of Hygiene and Tropical Medicine, the National Institute for Medical Research, Tanzania, Kilimanjaro Christian Medical College, and the Centre for Medical Parasitology, University of Copenhagen, for their participation in the community studies that underpin this analysis.

***Financial support.*** This work was supported by the UK Medical Research Council (grants G9901439, G0600718, and G0600230; grants MR/K000551/1, MR/M01360X/1, and MR/N010469 to T. G. C.; and grant MR/M01360X/1) to S. G. C.); the Danish International Development Agency; the Gates Malaria Partnership (Bill and Melinda Gates Foundation); the Malaria Genetic Epidemiology Network (MalariaGEN); the Wellcome Trust (grant WT077383/Z/05/Z to MalariaGEN; grant 090770/Z/09/Z to the Resource Centre for Genomic Epidemiology of Malaria; grants 075491 and 090532 as core support to the Wellcome Trust Centre for Human Genetics; and grant 091924 to C. J. D.); the Bill and Melinda Gates Foundation through the Foundations of the National Institutes of Health (566), as part of the Grand Challenges in Global Health Initiative and Fundação para Ciência e Tecnologia (grant UID/MAT/00006/2013 to N. S.).

***Potential conflicts of interest.*** All authors: No reported conflicts of interest. All authors have submitted the ICMJE Form for Disclosure of Potential Conflicts of Interest. Conflicts that the editors consider relevant to the content of the manuscript have been disclosed.
