# Supplementary figures and images for: Malaria Host Candidate Genes Validated by Association With Current, Recent, and Historical Measures of Transmission Intensity
Source: J Infect Dis. 2017 May 25;216(1):45–54. doi: 10.1093/infdis/jix250 (PMC5853769; doi:10.1093/infdis/jix250)

## Altitude

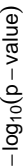

### Seroconversion rate

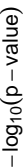

## Parasite rate

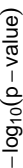

Supplement: Supplementary_Figure_2 [file jix250_suppl_supplementary_figure_2.pdf]

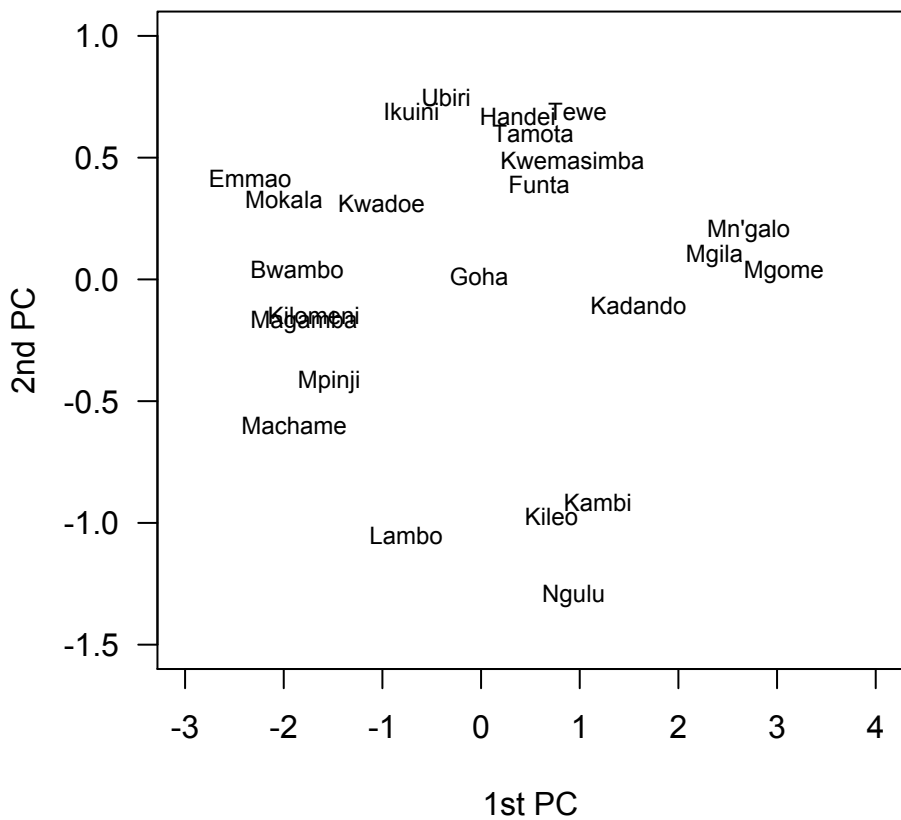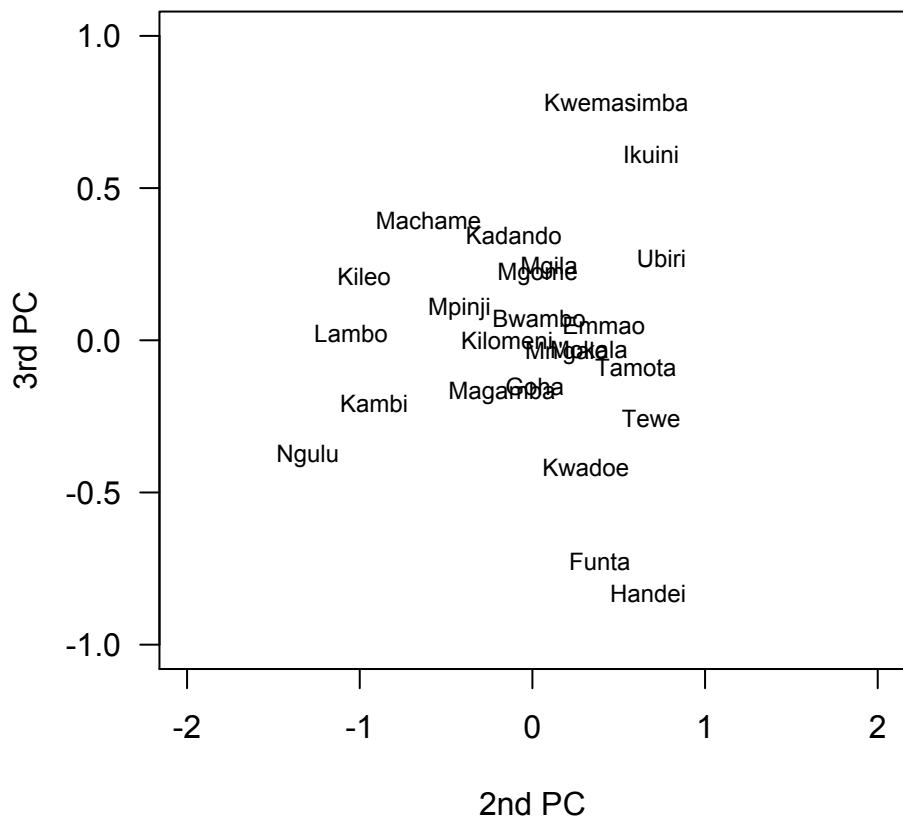

Supplement: Supplementary_Figure_3 [file jix250_suppl_supplementary_figure_3.pdf]
