# Supplementary material for: Malaria Host Candidate Genes Validated by Association With Current, Recent, and Historical Measures of Transmission Intensity
Source: J Infect Dis. 2017 May 25;216(1):45–54. doi: 10.1093/infdis/jix250 (PMC5853769; doi:10.1093/infdis/jix250)
Supplement: Supplementary_Table_1 [file jix250_suppl_supplementary_table_1.docx]

**Supplementary Table 1**

False discovery rates compatible with a threshold of 0.01 for each variable under analysis.

| **Variable under analysis** | **Range of false discovery rates** |
| --- | --- |
| Altitude | 0.08-0.15 |
| Seroconversion rate | 0.15-0.19 |
| Log odds of parasite rate | 0.08-0.11 |
| Principal component 1 | 0.11 |
| Principal component 2 | 0.14-0.15 |
