# Supplementary material for: Malaria Host Candidate Genes Validated by Association With Current, Recent, and Historical Measures of Transmission Intensity
Source: J Infect Dis. 2017 May 25;216(1):45–54. doi: 10.1093/infdis/jix250 (PMC5853769; doi:10.1093/infdis/jix250)
Supplement: Supplementary_Table_2 [file jix250_suppl_supplementary_table_2.docx]

**Supplementary Table 2**

Average correlation between malaria transmission measures using Spearman’s coefficient and corresponding mean coefficients on the first and second principal component axes after performing imputing data of α-thalassaemia genotype frequencies and seroconversion rate estimates that were missing.

| **Variables** | **Correlation matrix** | | |  | **Principal component analysis** | |
| --- | --- | --- | --- | --- | --- | --- |
|  | **Altitude** | **SCR** | **PR** |  | **1^st^ PC** | **2^nd^ PC** |
| Altitude | 1.00 | -0.791 | -0.688 |  | -0.601 | 0.191 |
| SCR | -0.791 | 1.00 | 0.562 |  | 0.578 | -0.562 |
| Parasite rate | -0.688 | 0.562 | 1.00 |  | 0.552 | 0.795 |
| Cumulative variance (%) | NA | NA | NA |  | 82.3 | 94.9 |
