# Supplementary material for: Malaria Host Candidate Genes Validated by Association With Current, Recent, and Historical Measures of Transmission Intensity
Source: J Infect Dis. 2017 May 25;216(1):45–54. doi: 10.1093/infdis/jix250 (PMC5853769; doi:10.1093/infdis/jix250)
Supplement: Supplementary_Figure_1 [file jix250_suppl_supplementary_figure_1.pdf]

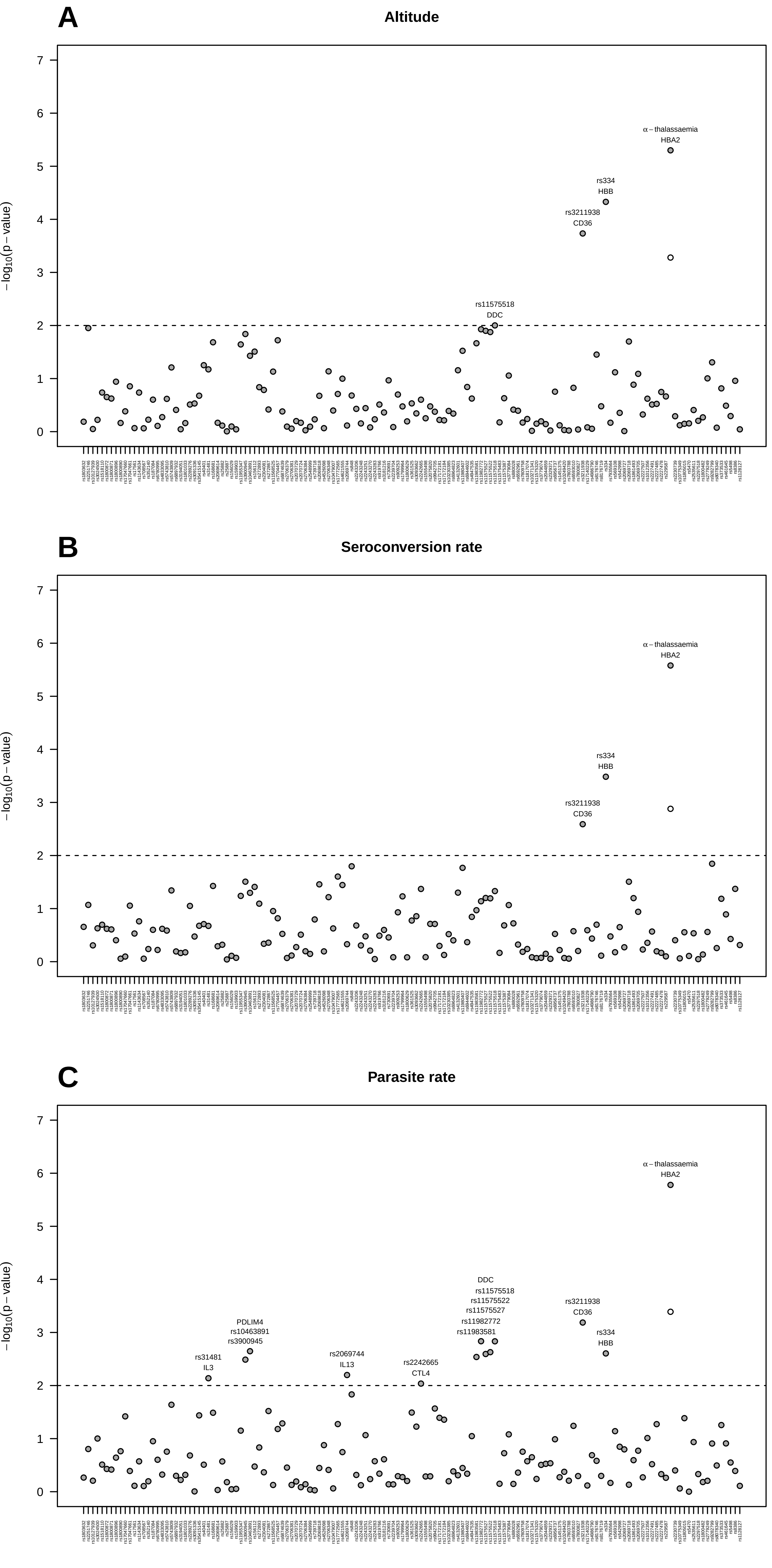
A
Altitude
−log10(p − value)
α − thalassaemia
HBA2
rs334
HBB
rs3211938
CD36
rs11575518
DDC
B
Seroconversion rate
−log10(p − value)
α − thalassaemia
HBA2
rs334
HBB
rs3211938
CD36
C
Parasite rate
−log10(p − value)
α − thalassaemia
HBA2
DDC
rs11575518
rs11575522
rs11575527
rs11982772
rs11983581
rs3211938
CD36
rs334
HBB
PDLIM4
rs10463891
rs3900945
rs31481
IL3
rs2069744
IL13
rs2242665
CTL4
